# Comparison Between Dexmedetomidine and Propofol for Sedation on Outcomes After Cardiac Surgery in Patients Requiring Mechanical Ventilation: A Meta-Analysis of Randomized-Control Trials

**DOI:** 10.7759/cureus.42212

**Published:** 2023-07-20

**Authors:** Lubna Sattar, Ibrahim Reyaz, Anurag Rawat, Raam Mannam, Abhimanyu Karumanchi, Venu Gopal Reddy Depa, Saima Batool, Muhammad Usama

**Affiliations:** 1 Medicine, Shadan Institute of Medical Sciences, Hyderabad, IND; 2 Internal Medicine, Christian Medical College and Hospital, Ludhiana, IND; 3 Interventional Cardiology, Himalayan Institute of Medical Sciences, Dehradun, IND; 4 General Surgery, Narayana Medical College, Nellore, IND; 5 Medicine, Kamineni Institute of Medical Sciences, Hyderabad, IND; 6 Internal Medicine, MNR Medical College and Hospital, Sangareddy, IND; 7 Internal Medicine, Hameed Latif Hospital, Lahore, PAK; 8 Neurology, Sheikh Zayed Medical College & Hospital, Rahim Yar Khan, PAK

**Keywords:** meta-analysis, cardiac surgery, post-operative outcomes, propofol, dexmedetomidine

## Abstract

The aim of this study was to compare outcomes between dexmedetomidine and propofol for sedation after cardiac surgery in patients requiring mechanical ventilation. This meta-analysis was performed according to Preferred Reporting Items for Systematic Reviews and Meta-Analyses (PRISMA) guidelines. Online databases, including EMBASE, PubMed, and the Cochrane Library, were comprehensively searched to identify relevant randomized controlled trials (RCTs) comparing the safety and efficacy of dexmedetomidine and propofol in patients undergoing cardiac surgery and requiring mechanical ventilation. The examined outcomes included the mean length of intensive care unit (ICU) stay in hours, duration of mechanical ventilation in hours, length of hospital stay in days, and number of patients diagnosed with delirium. A total of 14 studies were included in the present meta-analysis while 1360 patients undergoing cardiac surgery were involved in these studies. Pooled results showed that the duration of mechanical ventilation was lower in the dexmedetomidine group compared to the propofol group (mean difference (MD): 0.75, 95% confidence interval (CI): 0.06-1.44, p-value: 0.03). We also found a significantly low length of stay in ICU in the dexmedetomidine group compared to the propofol (MD: 0.89, 95% CI: 0.04-1.74, p-value: 0.04). The length of hospital stay was also significantly lower in patients receiving dexmedetomidine as compared to the propofol group (MD: 0.51, 95% CI: 0.32-0.70, p-value<0.001). Risk of delirium was significantly higher in patients receiving propofol compared to patients receiving dexmedetomidine (RR: 2.02, 95% CI: 1.48-2.74, p-value<0.001). In conclusion, our meta-analysis provides evidence of the beneficial impacts of dexmedetomidine on clinical outcomes in patients undergoing cardiac surgery. Dexmedetomidine was associated with a significant reduction in the duration of mechanical ventilation, length of stay in the intensive care unit (ICU) and hospital, and the risk of delirium.

## Introduction and background

Every year, more than two-million cardiac surgeries are performed worldwide [1]. Although cardiac surgery is commonly utilized to treat complications arising from ischemic heart disease, correct congenital heart disease, or address valvular heart disease caused by factors such as endocarditis, rheumatic heart disease, and atherosclerosis, these procedures come with several drawbacks [2]. The performance of cardiac surgery is known to carry significant risks of cardiovascular complications and other unfavorable outcomes, often resulting in extended hospital stays and even mortality [3-5]. Despite notable advancements in equipment, techniques, and medical care that have led to reduced rates of major complications and mortality [6-7], there remains a need for effective and safe perioperative medication to further minimize these adverse events [8].

While propofol is widely employed as a sedative agent in operating rooms and intensive care units (ICUs), it can potentially lead to hypotension, bradycardia, respiratory depression, and even apnea, depending on the dosage used for infusion [9-10]. On the other hand, dexmedetomidine is a selective agonist of α2 receptors. Its utilization in fast-track procedures is on the rise due to its advantageous properties, including sedation, analgesia, and anxiolysis, without causing respiratory depression. Dexmedetomidine induces a sedative effect that closely resembles natural sleep patterns when examined using electroencephalography, thereby preserving cognitive functions. Patients can be easily awakened, allowing for improved cooperation. However, it should be noted that depending on the infusion dosage, dexmedetomidine may result in hypotension and bradycardia [11-12].

An earlier meta-analysis attempted to elucidate the role of these sedatives in post-cardiac surgical sedation [13]. However, the study's findings were limited due to methodology issues and the inclusion criteria, as pointed out in a letter addressed to the editor [14]. Despite this, propofol was preferred to benzodiazepines for sedation among patients in the cardiac surgical ICU, according to the 2018 Clinical Practise Guidelines for the Prevention and Management of Pain, Agitation/Sedation, Delirium, Immobility, and Sleep Disruption in Adult Patients in the ICU (PADIS) guidelines [15]. However, there was a lack of sufficient data on the efficacy of dexmedetomidine versus propofol in this population. A meta-analysis conducted by Heybati et al. reported that the use of dexmedetomidine did not have a significant effect on the length of stay in the ICU when compared to propofol. However, it did lead to a significant decrease in the duration of mechanical ventilation and a reduced risk of delirium among patients undergoing cardiac surgery [16]. Nonetheless, this meta-analysis included both cardiac surgery patients and non-cardiac surgery patients. Furthermore, since the publication of this meta-analysis, certain new randomized control trials have been published that reported postoperative outcomes between patients who received dexmedetomidine and propofol. Therefore, we conducted this meta-analysis to compare outcomes between dexmedetomidine and propofol for sedation after cardiac surgery in patients requiring mechanical ventilation.

## Review

Methodology

This meta-analysis was performed according to Preferred Reporting Items for Systematic Reviews and Meta-Analyses (PRISMA) guidelines. Online databases, including EMBASE, PubMed, and the Cochrane Library, were comprehensively searched to identify relevant randomized controlled trials (RCTs) comparing the safety and efficacy of dexmedetomidine and propofol in patients undergoing cardiac surgery and requiring mechanical ventilation. The following Medical Subject Heading (MeSH) terms and free texts were utilized in different combinations to select eligible articles: "dexmedetomidine," "propofol," "cardiac surgery," "heart surgery," and "coronary artery bypass grafting," without placing restrictions on the year of publication. Our search was restricted to studies published in the English language only. To further expand the search, the reference lists of all included articles were manually searched. Two authors independently screened all records obtained from the online database search. The first-level screening was done based on titles and abstracts, followed by full-text screening. For this purpose, full texts of eligible records were obtained, and a detailed assessment was done based on predefined inclusion and exclusion criteria. Any disagreement in the study selection process was resolved through discussion.

Inclusion and Exclusion Criteria

According to the Participants, Interventions, Comparisons, Outcomes, and Study (PICOS) design protocol, the following criteria were used.

Participants: We included patients undergoing cardiac surgery, including coronary artery bypass grafting (CABG), aortic surgery, percutaneous coronary intervention, valve surgery, and others.

Intervention and comparison: Studies comparing dexmedetomidine and propofol.

Outcomes: Postoperative outcomes, including the mean length of ICU stay in hours, duration of mechanical ventilation in hours, length of hospital stay in days, and number of patients diagnosed with delirium.

Study design: Only randomized controlled trials (RCTs) were included in this meta-analysis to ensure the quality of pooled results. We excluded observational studies, reviews, editorials, and animal studies. Studies without a control group were also excluded.

Data Extraction

Two reviewers independently extracted the characteristics and relevant endpoint data from the included studies. The baseline information included details such as the first author, publication year, patient numbers in each group, average age, gender distribution, and type of cardiac surgery. The examined outcomes included the mean length of ICU stay in hours, duration of mechanical ventilation in hours, length of hospital stay in days, and number of patients diagnosed with delirium.

Quality Assessment

Two researchers independently evaluated the overall quality following the guidelines for quality assessment found in the Cochrane Handbook for Systematic Reviews of Interventions. In each included study, a number of quality issues were investigated, including selection bias, blinding bias, inadequate outcome data bias, selective reporting prejudice, and other possible biases. In the event of a dispute, a third investigator was brought in to settle the matter. The final quality was assigned one of three levels of bias risk based on the findings of the overall quality assessment: low, high, or unclear risk of bias.

Statistical Analysis

Mean and standard deviation were used to present the continuous data. A mean difference (MD) with a 95% confidence interval (CI) was used to compare continuous outcomes between the two groups. For dichotomous outcomes, a risk ratio (RR) with 95% CI was reported. Heterogeneity was assessed using the I-square statistics and Q test, with a p-value of less than 0.1 considered significant for heterogeneity. Based on the findings of the heterogeneity analysis, the random-effects model was employed when significant heterogeneity was reported. Conversely, if no significant heterogeneity was found, the fixed-effects model was utilized. The meta-analysis was performed using RevMan version 5.4.1 (The Cochrane Collaboration, London, United Kingdom).

Results

**Figure 1:**
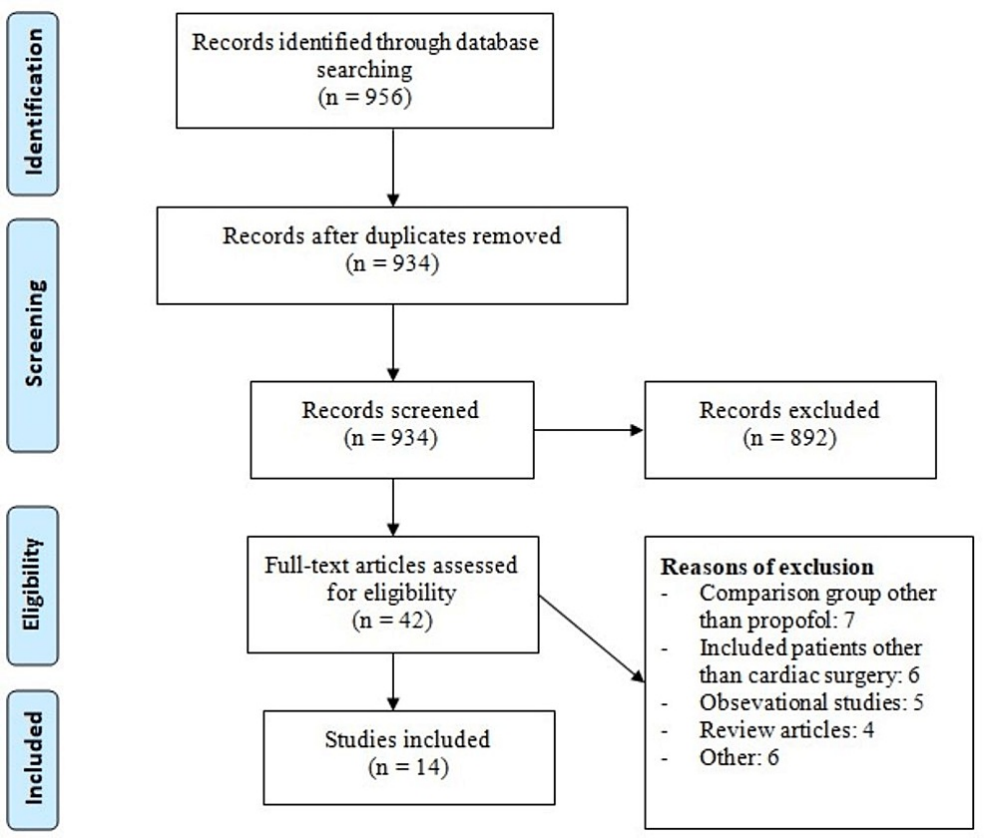
PRISMA flowchart of study selection PRISMA: Preferred Reporting Items for Systematic Reviews and Meta-Analyses

**Figure 2:**
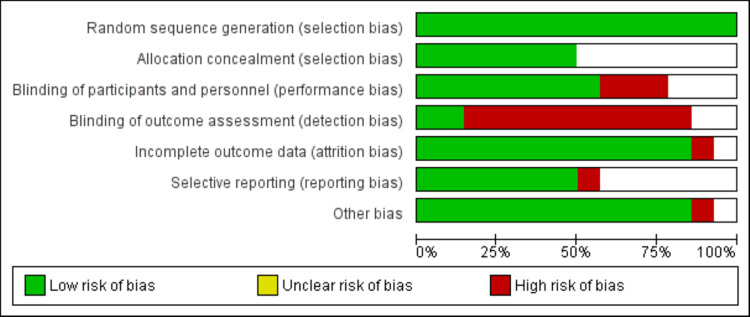
Risk of bias graph

Figure 1 shows the PRISMA flowchart of study selection. A total of 956 records were retrieved through online database searching and the studies were screened according to the predefined inclusion and exclusion criteria. Eight hundred ninety-two of these studies were excluded after the initial screening process using abstract and title. The full text of 42 studies was obtained and a detailed evaluation was done. Out of these studies, 14 studies fulfilled the eligibility criteria and were included in the present meta-analysis. A total of 1360 patients undergoing cardiac surgery were involved in these studies. The baseline characteristics of the included studies are shown in Table 1. Figure 2 shows the risk of bias assessment of all included studies.

**Table 1:** Characteristics of included studies CABG: coronary artery bypass surgery; CPB: cardiopulmonary bypass; NR: not reported

Author Name	Publication Year	Region	Surgery Type	Groups	Number of Participants	Mean Age (Years)	Males (%)
Abdallah et al [17]	2021	Egypt	CABG or valve replacement	Propofol	49	NR	NR
				Dexmedetomidine	49		
Corbett et al [18]	2005	United States	CABG	Propofol	46	62.4/ 63.6	82.6/ 81.4
				Dexmedetomidine	43		
Djaiani et al [19]	2016	United States	CABG or valve replacement	Propofol	92	72.4/ 72.7	76/ 74.7
				Dexmedetomidine	91		
Elgebaly et al [20]	2018	Egypt	Open heart surgery	Propofol	25	52.5/ 53.7	30/ 50
				Dexmedetomidine	25		
Eremenko et al [21]	2014	Russia	CABG or valve replacement	Propofol	27	NR	NR
				Dexmedetomidine	28		
Karaman et al [22]	2015	Turkey	CABG	Propofol	31	63.9/ 62.5	87.9/ 83.8
				Dexmedetomidine	33		
Liu et al [23]	2016	China	Elective cardiac surgery with CPB	Propofol	44	56.5/ 53	31.8/ 47.7
				Dexmedetomidine	44		
Maldonado et al [24]	2009	United States	Elective cardiac surgery with CPB	Propofol	30	58/ 55	58/ 65
				Dexmedetomidine	30		
Mogahd et al [25]	2017	United States	CABG	Propofol	35	54.8/ 53.4	57/51.4
				Dexmedetomidine	35		
Patil et al [26]	2021	India	CABG	Propofol	30	NR	NR
				Dexmedetomidine	30		
Sharaf et al [27]	2022	Egypt	Elective cardiac surgery	Propofol	75	68.9/ 67.9	50.7/ 52
				Dexmedetomidine	75		
Sheikh et al [28]	2018	India	Elective open heart surgery	Propofol	16	35.6/ 33.6	NR
				Dexmedetomidine	16		
Subramaniam et al [29]	2019	United States	CABG	Propofol	61	69/ 71	83.3/ 86.7
				Dexmedetomidine	59		
Susheela et al [30]	2017	United States	Elective cardiac surgery	Propofol	6	NR	NR
				Dexmedetomidine	6		

Meta-analysis of outcomes

Duration of Mechanical Ventilation (In Hours)

**Figure 3:**
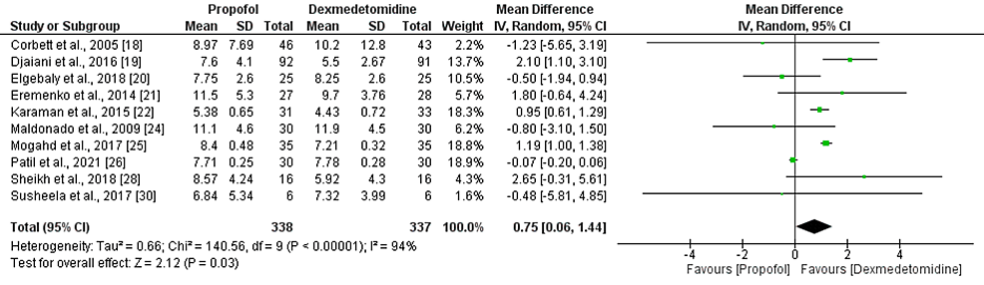
Duration of mechanical ventilation (hours) References [18-22,24-26,28,30]

A meta-analysis of 10 studies on dexmedetomidine versus propofol found that the duration of mechanical ventilation was significantly lower in the dexmedetomidine group compared to the propofol group (MD: 0.75, 95% CI: 0.06-1.44, p-value: 0.03) as shown in Figure 3. As significant heterogeneity was there across these studies (I-square: 94%, p-value: 0.03), the random effect model was used. A sensitivity analysis was performed to identify the source of heterogeneity, and the result showed that the study of Patil et al. [26] might be responsible for it, as excluding this study reduced heterogeneity from 94% to 48% as shown in Appendix A.

Length of Stay in ICU (In Hours)

**Figure 4:**
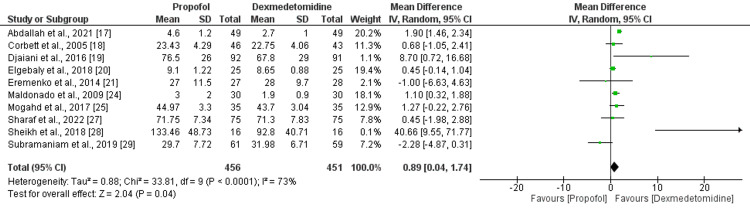
Length of ICU stay (hours) References [17-21,24-25,27-29]

Ten studies provided data on the length of stay in the ICU. Statistical heterogeneity was there among the studies (I-square: 73%, p-value<0.001). Combined results from the 10 RCTs of dexmedetomidine versus propofol found a significantly low length of stay in ICU in the dexmedetomidine group compared to the propofol (MD: 0.89, 95% CI: 0.04-1.74, p-value: 0.04) as shown in Figure 4. The sensitivity analysis was performed to identify the source of heterogeneity, and the result demonstrated that the study conducted by Abdallah et al., 2021, could be responsible for it [17].

Length of Hospital Stay (In Days)

**Figure 5:**
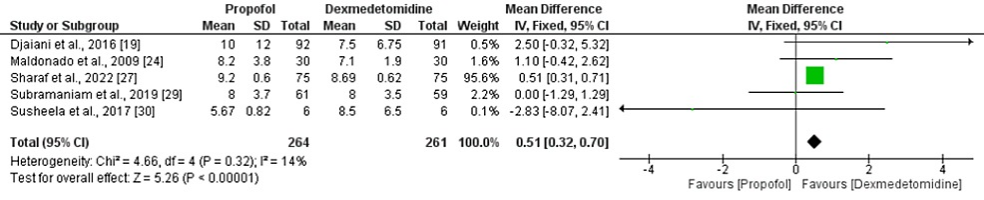
Length of hospital stay (in days) References [19,24,27,29-30]

Five RCTs compared the data on the length of hospital stay in patients undergoing cardiac surgery. No significant heterogeneity was reported among the study results (I-square: 14%, p-value: 0.32) and the fixed effect model was used. The combined results of five RCTs suggested that the length of hospital stay was significantly lower in patients receiving dexmedetomidine compared to the propofol group (MD: 0.51, 95% CI: 0.32-0.70, p-value<0.001) as shown in Figure 5. As most of the weight in this outcome was carried by Sharaf et al., 2022, we performed a sensitivity analysis by removing this study. As shown in Appendix B, the total hospital stay was lower in the dexmedetomidine group but the difference was insignificant.

Risk of Delirium

**Figure 6:**
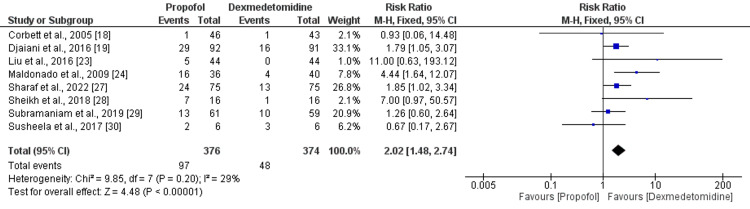
Risk of delirium References [18-19,23-24,27-30]

Eight studies were included in the pooled analysis of the risk of delirium. As shown in Figure 6, the risk of delirium was significantly higher in patients receiving propofol compared to patients receiving dexmedetomidine (RR: 2.02, 95% CI: 1.48-2.74, p-value<0.001). No significant heterogeneity was reported among the study results (I-square: 29%, p-value: 0.20).

Safety Events

We compared the risk of bradycardia and atrial fibrillation between two groups and the results are shown in Table 2. The risk of bradycardia was higher in the dexmedetomidine group compared to the placebo group but the difference was statistically insignificant. Similarly, the risk of atrial fibrillation was not significantly different between the two groups.

**Table 2:** Safety outcomes RR: risk ratio; CI: confidence interval

Outcome	RR (95% CI)	I-square
Bradycardia	0.32 (0.07-1.57)	0%
Atrial fibrillation	1.54 (0.59-4.02)	70%
Hypotension	0.59 (0.41-0.86)	0%

Discussion

Despite significant advancements in cardiac surgery that have reduced complications and mortality rates, there is still a need for effective drugs to benefit patients undergoing these procedures. Our meta-analysis indicates that dexmedetomidine may have beneficial effects on clinical outcomes in cardiac surgery patients, including reducing the duration of mechanical ventilation, length of stay in the intensive care unit (ICU) and hospital, and risk of delirium.

Our study found a significantly shorter duration of mechanical ventilation in the dexmedetomidine group compared to the propofol group. Similar findings were reported in a review by Heybati et al. [16]. Another study by Hu et al. demonstrated that patients who received dexmedetomidine were extubated three hours earlier compared to those given propofol [31]. These results align with the known sedative properties of dexmedetomidine, which promote consciousness, patient compliance, improved communication, and enhanced pain management. Dexmedetomidine's shorter duration of action and minimal impact on the urge to breathe contribute to these benefits, distinguishing it from propofol [32].

Our meta-analysis also revealed a shorter length of stay in the ICU and hospital for the dexmedetomidine group compared to the propofol group. Prolonged stays in the ICU and shorter hospital stays carry implications such as increased susceptibility to infections, unfavorable outcomes, and financial concerns [13]. Therefore, management plans should address these aspects as well.

In a recent meta-analysis of cardiac surgery patients, no significant reduction in the occurrence of delirium was observed [33]. However, when trials administering dexmedetomidine as adjuncts were excluded, a notable reduction in ICU delirium was seen among patients who received dexmedetomidine compared to propofol. Previous reviews have also concluded that dexmedetomidine sedation may lead to a lower incidence of ICU delirium compared to propofol [34-35]. Our high-certainty evidence from the meta-analysis demonstrated a significant reduction in the risk of ICU delirium in cardiac surgical patients who received dexmedetomidine. The majority of studies included in this meta-analysis used the confusion assessment method (CAM) to assess delirium. The precise mechanisms by which dexmedetomidine reduces the likelihood of delirium and the underlying pathophysiology of delirium are not fully understood. However, studies attribute this advantage to dexmedetomidine's sparing activity on gamma-aminobutyric acid receptors, minimal impact on respiration, ability to mimic normal sleep patterns, lack of anticholinergic activity, and potential to reduce the need for opioid medications [36-37].

The potential cardiovascular complications associated with dexmedetomidine sedation should be considered. The increased risk of bradycardia observed in patients undergoing cardiac surgery aligns with the findings reported by Abowali et al. [13]. This significant finding is consistent across patients undergoing medical procedures or other surgeries, and those with sepsis [16]. Our meta-analysis indicated a higher risk of bradycardia in patients receiving dexmedetomidine compared to the propofol group, although the difference was not statistically significant, potentially due to the limited number of studies assessing this outcome. While bradycardia has been reported with the use of dexmedetomidine, it can be effectively resolved with fluid boluses. Wu et al. also suggested a potential association between cardiovascular effects and high-dose dexmedetomidine infusion or the use of a loading dose in a previous review [38]. Therefore, close monitoring of patients in this specific subgroup, regardless of the sedation agent used, is advised, employing advanced techniques such as continuous heart rhythm and non-invasive blood pressure monitoring. The administration of boluses should also be approached with caution.

Our review included a larger number of trials with a more substantial patient sample, resulting in a comprehensive and updated evaluation of treatment effects compared to previous meta-analyses. Additionally, significant variability was observed in the dosage and duration of analgesic therapy administered during the post-surgical period, with inadequate documentation in some cases. Similar variability was noted in the use of other sedatives, such as benzodiazepines, during surgeries, indicating a lack of standardized protocols for general anesthesia. This variability may have influenced time-dependent outcomes, particularly delirium, which can be influenced by various contributing factors. Future trials should address these aspects and aim to follow standardized protocols. Furthermore, important outcomes like bradycardia and atrial fibrillation were not consistently assessed in the majority of studies, highlighting the need for future research to focus on the safety aspects of these drugs.

## Conclusions

In conclusion, our meta-analysis provides evidence of the beneficial impacts of dexmedetomidine on clinical outcomes in patients undergoing cardiac surgery. Dexmedetomidine was associated with a significant reduction in the duration of mechanical ventilation, length of stay in the ICU and hospital, and the risk of delirium. While dexmedetomidine is associated with potential cardiovascular complications, such as bradycardia, close monitoring of patients undergoing cardiac surgery is essential regardless of the sedation agent used. Advanced monitoring techniques, such as continuous heart rhythm and non-invasive blood pressure monitoring, should be employed to ensure patient safety.

## Figures and Tables

**Figure 7:**
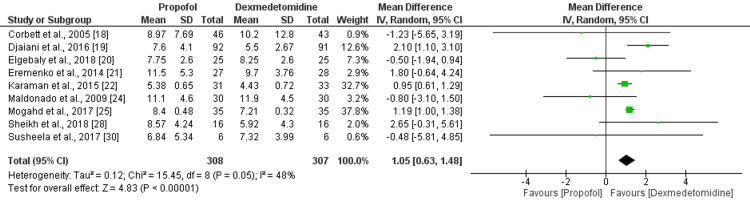
Forest plot comparison of mechanical ventilation after removing the study conducted by Patil et al.

**Figure 8:**
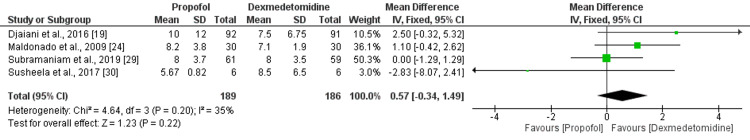
Forest plot comparison of the length of hospital stay after removing the study conducted by Sharaf et al.

## Appendices

Appendix A

Appendix B 
